# A Systematic Review of Factors Associated with Under-Five Child Mortality

**DOI:** 10.1155/2022/1181409

**Published:** 2022-12-05

**Authors:** Madhav Kumar Bhusal, Shankar Prasad Khanal

**Affiliations:** Central Department of Statistics, Tribhuvan University, Kirtipur, Kathmandu, Nepal

## Abstract

**Background:**

Preventing the life of the newborn and reducing the entrenched disparity of childhood mortality across different levels is one of the crucial public health problems, especially in underdeveloped and developing countries in the world. Sustainable development goals (SDGs)-3.2 is aimed at terminating all preventable under-five child mortality and shrinking it to 25 per 1000 live births or lower than this by 2030. Several factors have been shown to be linked with childhood mortality.

**Objective:**

This review is aimed at pointing out the significant determinants related to under-five child mortality by a systematic review of the literature.

**Methods:**

EMBASE, PubMed, Scopus database, and Google Scholar search engine were used for the systematic search of the literature. Special keywords and Boolean operators were used to point out the relevant studies for the review. Original research articles and peer-reviewed papers published in the English language till August 10, 2022, were included in the analysis and synthesis of the results. As per the Preferred Reporting Items for Systematic Review and Meta-Analysis (PRISMA) guidelines, out of 299 studies identified from different sources, only 22 articles were ascertained for this study. Eligible articles were appraised in detail, and relevant information was extracted and then integrated into the systematic review.

**Results:**

Mother's education, size of child at birth, age of mother at childbirth, place of residence, birth interval, sex of child, type of birth (single or multiple), and birth order, along with other socioeconomic, maternal, child, health facility utilization, and community level variables, were observed as important covariates of under-five mortality.

**Conclusion:**

Women's education and easy access to quality healthcare facilities should be the apex priority to lessen childhood mortality.

## 1. Introduction

Under-five mortality is a prime indicator of both sustainable development goals (SDGs) and millennium development goals (MDGs). The SDGs target 3.2 aims to end the preventable death of under-five mortality and to lower at least as low as 25 per 1000 live birth for all countries [1]. It is considered one of the major indicators to evaluate the level of child health, and it illuminates the overall development in countries. Under-five mortality rate enables the demographic assessment of the country's population and serves as a major indicator of the country's socioeconomic development and quality of life [2]. The MDG vision incepted in 2000 to fight against multidimensional poverty has remained a predominant development framework for the world by its termination in 2015. The global childhood mortality rate was 90 deaths per 1000 live birth in 1990. It declined by more than half during the MDG period to 43 deaths per 1000 live births in 2015; however, the target was to reduce by two-thirds between 1990 and 2015. The progress of 15-year MDGs shows a substantial decline in under-five child mortality and improvement in maternal health. Neonatal deaths occupied a higher proportion of under-five deaths in every region of the world. Preterm birth complications, complications during labor and delivery, and sepsis were reported as the major causes of these neonatal deaths. Reduction of neonatal deaths with some effective intervention is the major challenge to curtail under-five mortality [3].

The latest report by the United Nations Children's Fund (UNICEF) has shown that under-five mortality declined by 61 percent globally between 1990 and 2020 as there were 93 deaths per 1000 live births in 1990 to 37 deaths per 1000 live births in 2020. Despite this significant decline, the geographic disparity, economic variations among the countries, children in fragile contexts, and variation in sex-specific child mortality are the major issues of concern to overcome the high under-five child mortality. Moreover, the crisis of novel coronavirus (COVID-19) seems to have a serious impact on the efforts and interventions made to diminish childhood mortality. To cope with this adverse situation, more acceleration and attention are required to maintain and strengthen life-saving interventions [4]. Different socio-economic variables, maternal and child-related factors, and health service utilization factors like household wealth status; education level of the mother; older maternal age; use of polluting fuel; higher parity; lack of antenatal care (ANC) visits; lack of skilled birth attendance; use of antenatal iron and folic acid (IFA) supplementation; and tetanus toxoid (TT) vaccination during pregnancy were found major factors associated with childhood mortality by prior studies [5–10]. Moreover, some other studies have highlighted the role of community-level variables along with individual-level variables for the survival of children. These studies have suggested to include community-level variables in the study for a more accurate estimate and useful epidemiological understanding [11–15].

A proper understanding and identification of important determinants related to under-five child mortality are extremely essential to implement healthcare policies, effective allocation of healthcare resources, and to endorse appropriate interventions to reduce childhood mortality. This systematic review is thus aimed at assessing the factors associated with under-five mortality.

## 2. Methods

### 2.1. Data Sources and Search Strategy

Preferred Reporting Items for Systematic Review and Meta-Analysis (PRISMA) guidelines were followed to conduct this study [16]. Literature was searched systematically using different electronic databases including EMBASE, PubMed, and Scopus. In order to include the grey literature like government documents, reports, and working papers, the Google Scholar search engine was used. These computer-based searches were done up to August 10, 2022. The exhaustive published articles were retrieved from a specially designed search strategy. To retrieve the relevant articles from the databases as per the objective of this study, well-constructed keywords or phrases were formulated. The words were connected by “OR” and “AND” operators so that these operators enable to search the records in databases and search engines in an exclusive manner. The search strategy used was [(‘childhood mortality' OR ‘under-five mortality') AND (‘associated factors' OR ‘risk factors')]. The articles were searched by applying the search strategy in the title search in Scopus, PubMed, and Google Scholar. In the case of EMBASE, this search strategy was applied in the title or abstract search to extend the horizon of searches. This particular strategy was developed after multiple tests of various combinations of keywords in the database to include the majority of the literature related to exploring the factors associated with under-five mortality.

### 2.2. Study Selection

Articles selected from different databases and search engine were managed by using the open-source citation manager software Zotero (http://www.zotero.org). For this, all selected records were exported to Zotero from databases and search engine. Studies imported were then merged, and duplicate studies were eliminated after confirming if they consist same authors and the same research topic. After then, all articles were exported to Rayyan (rayyan.ai) for further screening. In this phase, all the records were categorized into two groups “include” and “exclude.” The inclusion and exclusion decisions were done based on the title and abstract assessment of the articles. Decisions for the very few records were made based on the title assessment of the article in case of the dearth of the abstract. Rayyan is a free web-based tool that facilitates systematic review [17]. It helps to screen each paper quickly by allowing it to tag the paper into an inclusion or exclusion category after a brief examination. All the records grouped in the inclusion category were then exported into Zotero, and then, the full text of each record was reviewed microscopically. Articles satisfying the inclusion criteria were finally decided to incorporate into the study. Any differences while examining the records were eliminated from the consensus between authors.

### 2.3. Inclusion and Exclusion Criteria

**Figure 1:**
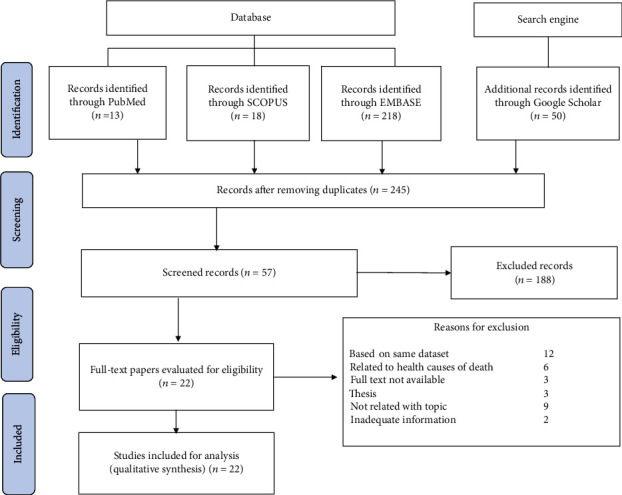
PRISMA flowchart for the selection of studies.

Some restrictions were created while selecting the papers to incorporate in the review. Articles were included for systematic review if they were (1) original research articles and grey literature matching the objective of the review; (2) peer-reviewed papers; (3) published in the English language; and (4) published till August 10, 2022. Studies were excluded if (1) the full text of the paper is not available; (2) the studies were thesis; (3) the studies were based on factors related to medical causes of under-five death; (4) the studies were not related to the objective of this study; (5) studies did not contain adequate information; and (6) published using the same dataset as published by other authors (s). The PRISMA flow chart for the selection process of the records is shown in Figure 1.

### 2.4. Data Extraction

All eligible papers were reviewed thoroughly to extract relevant information and were recorded into an excel sheet. The information extracted from each study was as follows: (1) first author of the study, (2) year of publication, (3) type of study design, (4) major method/technique used for analysis, (5) study area/region/country, (6) major objective of the study, (7) sample size/data source, and (8) findings (significant factors associated with under-five child mortality). MKB thoroughly extracted the data from each included record. In order to reduce selection bias and abate individual errors, SPK also verified the extracted information. Disagreement in the process of extracting the data between authors was solved by reassessing the article until having a common conclusion. The extracted data is shown in Table 1.

### 2.5. Quality Assessment

National Institute of Health (NIH) quality assessment tools for observational cohort and cross-sectional studies were used for the critical appraisal of each study [18]. For impartial judgment, both authors (MKB, and SPK) independently ranked the quality of each article. Any discrepancies in the course of appraisal were resolved by the consensus between authors. All studies lucidly stated their objectives and study population and had a participation rate of all eligible persons at least 50%. The subjects were selected from the same population in each study, and the determination of sample size was justified by all of them. Exposure of interest was not measured prior to outcome by 21 (95.45%) cross-sectional studies whereas exposure of interest was measured by one (4.55%) cohort study, and it had a sufficient timeframe to observe an association between exposure and outcome variables. The relation between different levels of exposure and outcome was examined by each study. Exposure measures were clearly defined but not assessed more than once by all studies. The outcome variable was clearly defined in each study, and the confounding variables were adjusted by all of them. None of the studies reported outcome assessors blinded to the exposure status of participants. The loss to follow-up after baseline was not applicable for 21 (95.45%) cross-sectional studies, while it was not reported in one (4.45%) cohort study. Additional information on quality assessment is included in the supplementary file.

### 2.6. Synthesis of the Results

An explicit descriptive summary of all the extracted records was prepared for the synthesis of the results. For this purpose, Table 1 is constructed. It contains different components including the name of the first author, year of publication, the objective of the study, data source and sample size, and findings. Major findings of included studies were assessed in a descriptive way from the extracted information to point out the significant determinants of under-five mortality.

## 3. Results

### 3.1. Search Findings

The purpose of this study is to explore the factors associated with under-five child mortality. Figure 1 shows the selection procedure and exclusion of records with specific reasons for rejection. Of the total 299 records identified from different databases and a search engine, 245 remained after removing 54 duplicate records. Of the 245 records, 188 were discarded after evaluating the title and abstract. Only 57 records remained for full-text analysis. After the detailed assessment of these 57 records, only 22 articles were found eligible for systematic review satisfying the inclusion criteria. The 35 records were discarded from the analysis for different reasons. Out of 35, 12 studies were removed due to the reason of using the same dataset as used by other studies, but one most comprehensive study based on such dataset is included in this review. Six studies were found concentrated to explore the causes of under-five mortality and related to medical reasons like lower respiratory infections, infections, preterm birth complications, nutrition, and diarrhea. These articles were removed from this review. Likewise, nine records included in the selection process were found irreverent to achieve the objective of this study. Such studies which only assessed the relationship between the socioeconomic status of the family and under-five mortality, assessing the factors associated with under-five mortality before and after the health campaign made by a community group, and studies conducted to measure under-five mortality across spatial units were excluded. Further, two records that did not contain sufficient information were also omitted from this review. One such discarded study was restricted to the factors allied to under-five mortality of boys and girls separately, and another study was limited to maternal factors only.

### 3.2. Characteristics of Included Studies

**Figure 2:**
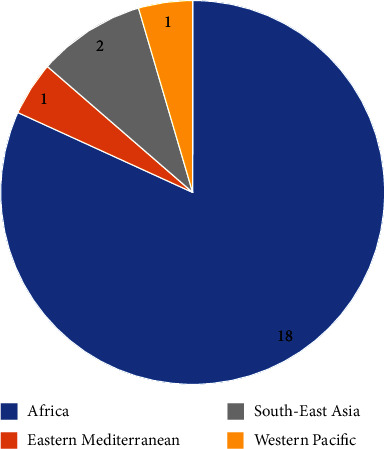
Studies included in systematic review from different regions (*n* = 22).

As shown in Figure 2, 22 eligible studies (listed in Table 1) were selected for systematic review from four different regions, namely, Africa, South-East Asia, Eastern Mediterranean, and Western Pacific.

Out of 22 studies, 18 studies were conducted in 11 countries in the Africa region (Burkina Faso [19–21]: 3, Ethiopia [22–24]: 3, Nigeria [25–27]: 3, Ghana [28, 29]: 2, Lesotho [30]: 1, sub-Saharan (combine study of five-country; Chad, Democratic Republic (DR) of Congo, Mali, Niger, and Zimbabwe) [31]: 1, Zambia [32]: 1, Kenya [33]: 1, DR Congo [34]: 1, Sierra Leone [35]: 1, and Tanzania [36]: 1). One study included was conducted in Nepal [37] and another one in Bangladesh [38] from South-East Asian region. Only one article was eligible to be included in this review from Pakistan [39], the Eastern Mediterranean region, and the next study selected was from Cambodia [40] from the Western Pacific region.

### 3.3. Summary of the Results

**Figure 3:**
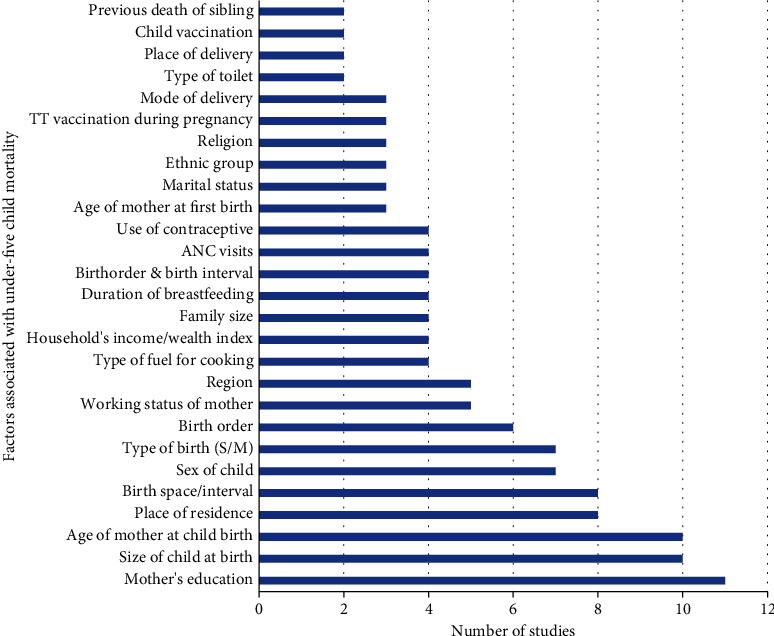
Number of studies and factors associated with under-five child mortality identified from studies (*n* = 22).

Various significant determinants of under-five child mortality were observed from a comprehensive review of articles.  Figure 3 shows the factors associated with under-five child mortality observed from at least two studies in the review. Out of 22 articles reviewed for the analysis, 11 (50%) articles reported that education of mother is a principal determinant of under-five mortality. Size of child at birth, age of mother childbirth, place of residence whether family resides in the rural or urban area, and birth space/interval are prime variables determining the survival of under-five children. Sex of child, type of birth (singleton or multiple/twin), birth order, working status of mother, region, type of fuel for cooking, household's wealth index, family size, duration of breastfeeding, birth order and birth interval, ANC visits, use of contraceptive, age of mother at first birth, marital status of mother, ethnic group, religion, TT vaccine taken during pregnancy, and mode of delivery were highly influential to determine the childhood mortality.

### 3.4. Thematic Distribution of Factors Related to Under-Five Child Mortality

**Figure 4:**
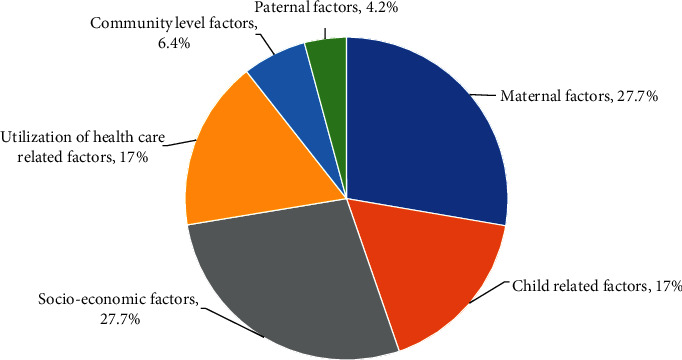
Percentage distribution of thematic factors related to under-five child mortality (*n* = 47).

Various factors associated with under-five child mortality extracted from the review are listed in the last column of Table 1. The micro-level scrutiny and analysis of reviewed articles provided 47 different factors responsible for under-five child mortality. These factors are distributed in six distinct thematic groups, namely, socio-economic factors, maternal factors, utilization of healthcare related factors, child related factors, and community level factors, as shown in Figure 4. The details of thematic factors are depicted in Table 2.

Maternal and socioeconomic factors were found equally and highly important to measure under-five child mortality. Out of 47, 13 (27.7%) factors were socioeconomic and maternal factors. The next two equally 8 (17%) of the total significant thematic factors were child related and utilization of healthcare related factors. Similarly, 3 (6.4%) and 2 (4.2%) factors out of 47 were community level and paternal factors, respectively.

#### 3.4.1. Factors Associated with Under-Five Child Mortality in the Studies Conducted in the Africa Region

**Figure 5:**
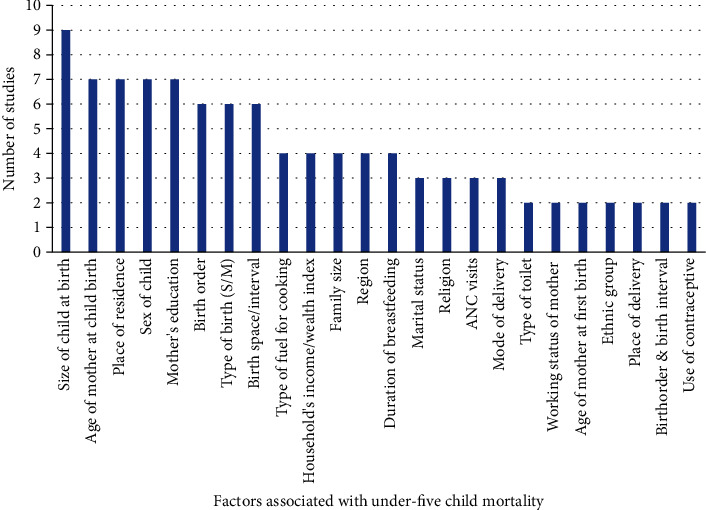
Number of studies and factors related to under-five mortality in Africa region (*n* = 18).

Figure 5 shows factors identified by two or more studies conducted in African countries. Among 18 articles, 9 (50%) articles [20, 24–26, 29, 31, 32, 35, 36] found that the size of child at birth is a major determinant playing an important role in under-five mortality. Age of mother, place of residence, sex of child, and mother's education are other important variables explored by 7 (38.9%) studies conducted in these countries. Similarly, birth order, type of birth, birth interval, type of fuel for cooking, household's wealth index, family size, religion, duration of breastfeeding, marital status, religion, ANC visits, and mode of delivery are also key variables to measure the under-five mortality.

#### 3.4.2. Factors Related to Under-Five Child Mortality in the Studies Conducted in the South-East Asia Region

**Figure 6:**
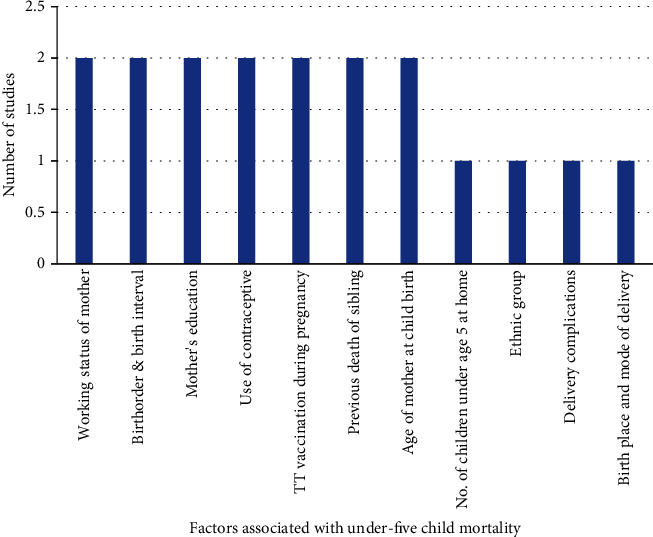
Number of studies and factors related to under-five mortality in South-East Asia region (*n* = 2).

Figure 6 exhibits the maternal, child-related, health service utilization, and socioeconomic covariates of under-five child mortality in the studies conducted in Asian countries.

Out of two studies [37, 38] conducted in this region, both studies explored that working status of the mother, birth order and birth interval, mother's education, use of contraceptives, TT vaccination during pregnancy, previous death of siblings, and age of mother at childbirth as significant determinant are associated with under-five mortality. The number of children under age 5 at home, ethnic group, delivery complication, and birthplace and mode of delivery were also obtained as important covariates in this region.

#### 3.4.3. Factors Related to Under-Five Child Mortality in the Study Conducted in the Eastern Mediterranean Region

Only one study [39] selected in this review was conducted in Pakistan, an Eastern Mediterranean country. Mother's education, whether she is educated or not; working status of the mother, whether she is involved in some work or not; birth interval, mother's age at first birth and size of child at birth, whether the weight of the child at birth was average or small are the important factors related to under-five child mortality obtained by this study.

#### 3.4.4. Factors Related to Under-Five Child Mortality in the Study Conducted in the Western Pacific Region

One study conducted in Cambodia [40] representing from Western Pacific region studied the determinants of under-five mortality using 2010 and 2014 Cambodian demographic and health survey datasets. It has explored that type of birth whether the birth was single or multiple, birth interval, age of mother at childbirth, mother's education, place of residence whether the family stay in rural or urban areas, region, ANC visits, dose of TT vaccine received, and status of child vaccination whether the child was fully vaccinated or not are the major covariates of under-five mortality.

#### 3.4.5. Common Factors Related to Under-Five Child Mortality in the Studies Conducted in African, South-East Asia, Eastern Mediterranean, and Western Pacific Regions

**Figure 7:**
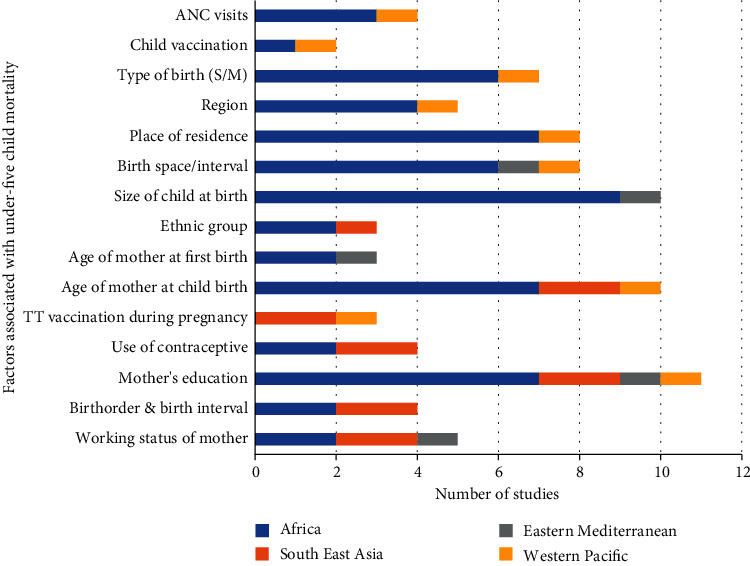
Number of studies with common factors affecting under-five child mortality in different regions (*n* = 22).

Among the significant determinants of under-five child mortality observed in this review, only one determinant has appeared common in all four regions while some other determinants appeared common only in two and three regions as depicted in Figure 7. The education of the mother was observed as a common significant covariate in all four regions. Out of 18 studies carried out in African countries, 7 (38.9%) studies [23–25, 27, 29, 31, 36] have reported that a mother's education is a significant determinant of under-five child mortality while both the studies conducted in South-East Asian countries [37, 38] and one study [39] conducted in each of Eastern Mediterranean and Western Pacific region [40] found education of mother as a significant covariate of under-five mortality. Working status of the mother was obtained as a common covariate of under-five mortality in Africa, South-East Asia, and Eastern Mediterranean regions. Likewise, the variables age of the mother at childbirth were found common in three regions except for the Eastern Mediterranean region while birth interval appeared common except in the studies conducted in countries of the South-East Asian region. Birth order and birth interval, use of contraceptives, and ethnic group of mothers were observed as common covariates of under-five mortality in Africa and South-East Asian regions. TT vaccine taken during pregnancy is another important covariate reported by the two studies conducted in South-East Asian countries and one study conducted in a country belonging to the Western Pacific region. The size of the child at birth and the age of mother at first birth are the next equally important variables reported by the studies conducted in African countries and one study conducted in Pakistan, belonging to the Eastern Mediterranean region.

Similarly, other significant common factors explored by the studies conducted in different African countries and one study carried out in Cambodia from the Western Pacific region were place of residence, region, type of birth whether the birth was single or multiple, child vaccination, and the number of ANC visits.

#### 3.4.6. Uncommon Factors Related to Under-Five Child Mortality in the Studies Conducted in African, South-East Asia, Eastern Mediterranean, and Western Pacific Regions

**Figure 8:**
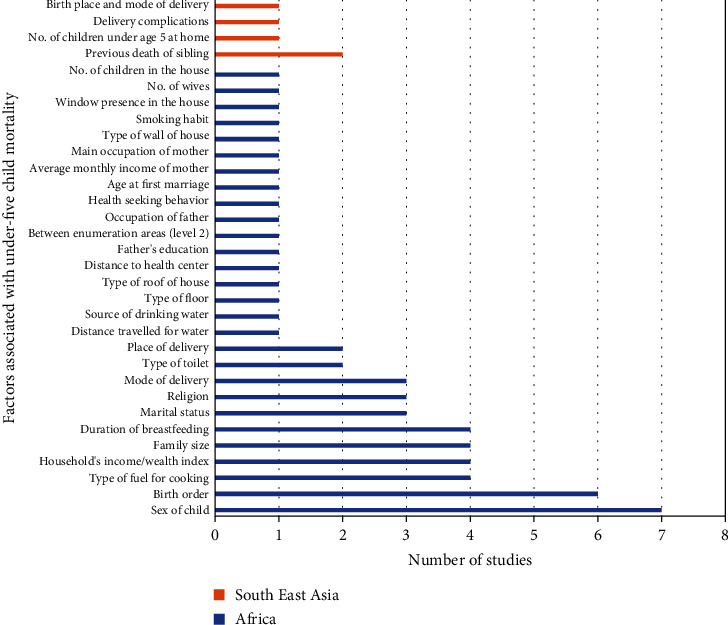
Number of studies with factors affecting the under-five child mortality separately in African and Asian countries (*n* = 22).

In contrast to the factors that appeared common and significant at least in two regions as discussed above, interestingly, various factors were found uncommon and appeared significant to explain under-five child mortality in the studies conducted in the same regions.  Figure 8 shows the several socioeconomic, maternal, paternal, child, and community level determinants of childhood mortality in different studies.

Sex of child, birth order, type of fuel for cooking, household's wealth index, family size, duration of breastfeeding, marital status, religion, mode of delivery, type of toilet, and place of delivery were obtained as significant determinants of under-five child mortality along with other factors as shown in Figure 8 in the studies carried out only in the African countries, whereas previous death of sibling, number of children under age 5 at home, delivery complications, and birthplace and mode of delivery were found important to explain childhood mortality only in the studies conducted in South-East Asian countries [37, 38].

## 4. Discussion

A large body of literature is available to explain the relationship between the survival of under-five children and its determinants around the globe. Our study examined the results of such 22 studies selected from a systematic procedure and obtained several significant covariates associated to under-five child mortality. We have observed that education of the mother, size of child at birth, age of mother at childbirth, place of residence, and birth interval are the predominant factors of under-five mortality along with other covariates. Moreover, some factors appeared common irrespective of the regions the studies were conducted, while some covariates were not found common in the studies conducted in different regions but were observed as important determinants of under-five child mortality.

The education of mothers appeared as a key factor to reduce under-five mortality [23–25, 27, 29, 31, 36–39]. Educated mother seems to be highly sensible and aware of the importance of health care utilization, nutrition, and sanitation to improve the health of child than their counterpart. Moreover, it is observed that even after controlling the family socioeconomic status, lower maternal and paternal education are both risk factors for under-five mortality [41, 42]. The size of the child at birth is another important risk factor for childhood mortality. Previous studies have shown significantly less relative odds of under-five death among children whose sizes are average or above at birth as compared to those whose sizes are small at birth [29, 36]. This fact implies the necessity of a balanced diet for the mother to improve the nutritional status of the child which ultimately helps to have a normal size of child. The age of mother at childbirth is another protective variable responsible for the survival of the child. Poor biological and social mechanisms at young age mothers have an adverse effect on the health of their first child. A child born to adolescent mothers exhibits fragile health outcomes and leads to a higher risk of under-five death [43]. Different studies [19, 32, 33] have shown that younger age (generally below 20 years) of mother at childbirth revealed significantly higher odds of under-five mortality with reference to middle age (20-34) of childbearing; however, there is no particular common age to segregate. Another prime dominant factor of childhood mortality is the place of residence. The rural area exhibited a higher risk of under-five mortality than the urban area in many studies [22, 25, 31, 33, 34, 40]. The significant disparity between rural and urban child mortality shows the immediate need for healthcare interventions and exploration of its causes. Substantial variation in rural-urban child mortality across socio-economic, biodemographic, and proximate factors was observed in a review study conducted using data from 35 (sub-Saharan) countries. To safeguard the survival of children, particularly in rural areas, it is paramount to provide easy access and quality healthcare services and to strengthen maternal and child health programs [44]. The next significant covariate of under-five mortality is birth space. Prior studies have shown that short birth intervals and child survival are inversely related. Women with short birth intervals possess higher odds of under-five mortality [45, 46]. Among various possible causes, this significant association may exist because a mother within the short birth interval (≤ to 18 months) could not be fit biologically for subsequent birth due to loss of nutrients and blood loss during breastfeeding and preceding pregnancy, respectively. Moreover, there is a higher possibility of facing different obstetrics complications in those mothers with short birth space as compared to those who have long birth intervals [47]. Type of birth, whether it was a singleton or multiple, also affects the health of a newborn. Different studies have revealed that multiple births have an inverse association with under-five mortality [19, 20, 23, 30–32, 40]. Such a relationship may exist due to the consequences of many reasons. Some prevalent reasons are poor management of multiple births, higher possibility of birth defects in multiple births, higher risk in pregnancy in comparison to single birth, multiple births may cause growth retardation or premature birth, and other delivery complications [48, 49].

In different studies carried out to assess the determinants of child survival, birth order also emerged as another leading variable. The first-born child and child born with order four and above (however differ from one study to another) exhibit a higher risk of mortality in comparison to those in the middle. The association between birth order and survival of a child is found to be influenced by other variables, especially by birth spacing, age of mother, and variations in the family [23, 49, 50]. Employment of women is one of the important components of their empowerment, to make them financially independent and for the recognition of gender roles and gender relations. But many studies have shown a higher risk of under-five mortality in those mothers who are employed [22, 37–39]. Such a result indicates that there should be feasible child-care alternatives for working women instead of discouraging them to work. The regional disparity of under-five mortality is another important issue to be solved. Different studies found a significant relationship between regions and under-five mortality [20, 22, 32, 33, 40]. Such discrepancies in under-five mortality across the region could be due to uneven access to healthcare facilities or there might have different levels of childhood survival programs, policies, and interventions. It is imperative to explore the reasons and develop intervention strategies in order to reduce the gap. Many studies have revealed the inverse relation between the economic status of a family and under-five mortality. Poor families are compelled to have higher risks of under-five mortality compared to rich families [31, 39]. This disparity might exist due to several reasons. Families with poor economic status become unable to afford for expensive health care services in need, they may not provide sufficient nutritional foods for mothers and may be ignorant about the overall health care of child and mother, etc.

Family size is another influential variable of child survival. The study conducted to explore the risk factors for childhood mortality in sub-Saharan Africa found lower mortality risks for those who were born in large households [21], whereas another study observed higher infant mortality in those households having a large number of children (3-5 and ≥6) in comparison to those having 1-2 children [51]. These two findings imply that rather than the number of family members, a large number of children could be a detrimental factor in child mortality. The duration of breastfeeding is also a significant covariate of under-five child mortality. Past studies have shown that a longer period of breastfeeding reduces the risk of under-five mortality [20, 26, 30, 33]. Breastfeeding yields sufficient natural nutrition to the newborn and protects them from different ailments, and it also enhances the immune system of children [52]. ANC is a maternal health care program offered by trained health workers to pregnant women. Its main objectives are to recognize the risk, prevention, and control pregnancy-related diseases. It also offers health education for mothers and children [53]. Results from earlier studies revealed that an increase in the number of ANC visits reduces under-five mortality [23, 24, 32, 40]. This finding suggests implementing appropriate intervention programs to encourage ANC visits in order to significantly reduce under-five mortality. The use of contraceptives is an important measure to reduce childhood mortality. The likelihood of under-five mortality decreases with the use of contraceptives [23, 26, 37, 38]. Its uses increase the successive birth interval and contribute to increasing the survival of mothers and reducing childhood mortality [54].

This study contains some limitations. Although the principal objective of this study is to explore the exhaustive determinants of under-five mortality, the factors associated with medical causes responsible for under-five mortality are overlooked in this review. Identification of such factors besides socio-economic, maternal, child-related, health care utilization, and community level variables may provide more comprehensive information in the effort making to mitigate under-five mortality. Furthermore, the prior studies which were not available in the database and were not accessible in the exploration through search engines are excluded from this study. The results and interpretations made in this review could be different if such studies were included in the analysis. Also, the possible discrepancies in the factors explored by reviewed articles as a consequence of the application of distinct statistical models for a particular study design are disregarded in this study. Despite these limitations, this study, to the best of our understanding is an up-to-date systematic review to identify the factors associated with childhood mortality. The rigorous and meticulous review process followed to ascertain the factors of childhood mortality provided the list of significant covariates of childhood mortality. Such findings are expected to be helpful to formulate effective healthcare policies and introduce interventions in order to reduce child mortality. Further, the results would be a valuable reference for planning new studies based on primary data to explore the most promising factors associated with under-five child mortality and to quantify their effects on childhood mortality.

## 5. Conclusion

Reducing childhood mortality and improving maternal and child health is a key universal health problem. The efforts made especially in the extension of healthcare programs and facilities in the last few decades showed a significant reduction in childhood mortality. However, the existence of remarkable variations in childhood mortality across different levels still possesses a common prevailing challenge in front of all nations. We have extracted different socioeconomic, maternal, child-related factors allied to healthcare utilization and community-level variables as important determinants of under-five mortality. Education of mother, size of child at birth, age of mother at childbirth, place of residence, and birth interval were the significant and most frequently observed covariates of under-five mortality.

## Figures and Tables

**Table 1:** Characteristics of included studies (*n* = 22).

First author	Year	Study design	Method(s)	Study area	Objective(s)	Data source and sample size	Factors associated with under-five child mortality
Abir [38]	2015	Cross-sectional	Multilevel models	Country-wide (Bangladesh)	To identify factors associated with mortality in children under 5 years of age	Bangladesh DHS 2004, 2007, and 2011 (combine) (*n* = 16,722 live births)	Working status of mother; working (AOR = 1.67, 95%CI = 1.34, 2.08, *p* value < 0.001), maternal highest level of education; secondary or more with Ref to no education (AOR = 0.41, CI = 0.26, 0.63; *p* value < 0.001), previous death of siblings; yes (AOR = 6.00, CI = 4.28, 8.40; *p* value < 0.001), no. of children under age 5 at home; 3 and above (AOR = 0.11, CI = 0.08, 0.15, *p* value < 0.001), TT vaccination at pregnancy; one TT with Ref to never (AOR = 0.74, CI = 0.56, 0.96; *p* value = 0.023), 2 and more TT (AOR = 0.53, CI = 0.42, 0.66, *p* value < 0.001), delivery complications; any complications with Ref to none (AOR = 0.66, CI = 0.53, 0.84, *p* value = 0.001), contraceptive use; yes (AOR = 0.33, CI = 0.27, 0.40, *p* value < 0.001), Mother's age at child birth; 30-39 with Ref to <20 (AOR = 1.64, CI = 1.01, 2.65, *p* value = 0.046), birth rank and birth interval; 2^nd^/3^rd^ birth rank, ≤2 years interval with Ref to 2^nd^/3^rd^ birth rank, ≤2 years interval (AOR = 2.18, CI = 1.48, 3.21, *p* value < 0.001), 4^th^ birth rank, ≤2 years interval (AOR = 2.73, CI = 1.76, 4.23, *p* value < 0.001), birth place and mode of delivery; home with Ref to health facilities without caesarean (AOR = 0.58, CI = 0.41, 0.82, *p* value = 0.002)

Andegiorgish [32]	2022	Cross-sectional	Multilevel regression model	Country-wide (Zambia)	To describe the burden, trend, and associated factors of under-five mortality rate	Zambia DHS 2007, 2013-14, and 2018 (combine) (*n* = 29,274 live births)	Maternal age; 20-24 with Ref to 15-19 years (AOR = 0.74, *p* value < 0.05), 25-29 years (AOR = 0.61, *p* value < 0.01), 30-34 years (AOR = 0.63, *p* value < 0.05), wealth index; poorer with Ref to richest (AOR = 0.73, *p* value < 0.05), place of residence; rural with Ref to urban (AOR = 0.79, *p* value < 0.001), ANC visits; no ANC as compared to had at least one ANC visits (AOR = 3.17, *p* value < 0.001), birth type; multiple birth with Ref single (AOR = 2.54, *p* value < 0.001), size of child at birth; below average with Ref to average (AOR = 1.78, *p* value < 0.001), child sex; male with Ref to female (AOR = 1.28, *p* value < 0.001), regions; eastern with Ref to central (AOR = 1.52, *p* value < 0.01) Luapula (AOR = 1.50, *p* value < 0.01) Muchinga (AOR = 1.43, *p* value < 0.01)

Arku [28]	2016	Cross-sectional	Indirect demographic method and Bayesian spatial model	Country-wide (Ghana)	To estimate the under-five child mortality and its social and environmental risk factors	110 districts (10% random sample of Ghana's 2000 and 2010 National Population and Housing Census), and indirect demographic methods and Bayesian spatial model was used to estimate under-five mortality	Use of LPG in household for cooking (RR = 11.1%, 95%CI = 3.0%, 18.8%) associated with lower under-five mortality.

Ayele [22]	2015	Cross-sectional	Structured additive logistic regression model	Country-wide (Ethiopia)	To estimate the under-five mortality risk factors	Ethiopian DHS 2011 (*n* = 26,370 live births)	Distance to fetch water; 31-60 min. with Ref to on the premises (AOR = 1.076, CI = 1.030, 1.086) more than 60 min. (AOR = 1.096, CI = 1.013, 1.199), source of drinking water; tap water with Ref to unprotected water (AOR = 0.941, CI = 0.841, 0.986), cooking fuel; electricity/gas with Ref to straw/animal dung (AOR = 0.920, CI = 0.811, 0.986), type of toilet; no toilet facility with Ref to toilet with flush/pit latrine (AOR = 1.037, CI = 1.005, 1.157), other toilet type (AOR = 0.969, CI = 0.850, 0.998), type of floor; cement with Ref to wood (AOR = 1.281, CI = 1.072, 1.392), earth/sand/dung (AOR = 1.345, CI = 1.044, 1.466), type of roof; corrugated iron/metal with Ref to thatch/leaf/mud (AOR = 0.996, CI = 0.886, 0.998), mat/plastic sheet/wood (AOR = 0.998, CI = 0.979, 0.998), type of wall; cane/trunk/bamboo with Ref to wood planks/shingles (AOR = 0.811, CI = 0.801, 0.934), smoking habit; no (AOR = 0.807, CI = 0.601, 0.987), region, place of residence; rural (AOR = 1.094, CI = 1.014, 1.099), working status of mother; no (AOR = 0.957, CI = 0.837, 0.995), sex of child; female (AOR = 0.787, CI = 0.747, 0.971).

Becher [19]	2009	Cross-sectional	Penalized splines and Cox regression model	Country-wide (rural Burkina Faso)	To investigate the effect of multiple risk factors for childhood mortality	Nouna DSS, (children born alive between Jan 1, 1998, and Dec 31, 2001, and mortality follow-up to five years) (n =8,986 live births)	Sex of child; male (HR = 1.14, *p* value = 0.04), ethnic group; Peulh with Ref to Bwaba (HR = 1.39, *p* value = 0.03), religion; natural/other with Ref to Muslim (HR = 1.43, *p* value = 0.02), type of birth; twin with Ref to single (HR = 1.85, *p* value < 0.01), age of mother at birth; young (<18 years) with Ref to middle (18-34) (HR = 1.29, *p* value < 0.01), distance to next health center; >10 km (HR = 1.39, *p* value < 0.01).

Conombo [20]	2017	Cross-sectional	Logistic regression	Country-wide (Burkina Faso)	To examine the effects of risk factors on under-five mortality	Burkina Faso DHS-2010 (*n* = 15,044 live births)	Preceding birth interval; 18-23 with Ref to <18 months (OR = 0.56, 95%CI = 0.36, 0.88, *p* value = 0.011), 24-29 (OR = 0.43, CI = 0.28, 0.65, *p* value < 0.001), 30-35 (OR = 0.35, CI = 0.23, 0.54, *p* value < 0.001), 36-41 (OR = 0.27, CI = 0.17, 0.43, *p* value < 0.001), 42-47 (OR = 0.19, CI = 0.11, 0.33, *p* value < 0.001), 48-53 (OR = 0.26, CI = 0.14, 0.50, *p* value < 0.001), breastfeeding; never breastfeed with Ref to ever (OR = 2.89, CI = 1.96, 4.26, *p* value < 0.001), type of birth; twin with Ref to single (OR = 3.75, CI = 2.78, 5.06, *p* value < 0.001), birth order; 2 with Ref to 1 (OR = 0.41, CI = 0.21, 0.80, *p* value = 0.009), 3 with Ref to 1 (OR = 0.44, CI = 0.22, 0.91, *p* value = 0.026), size at birth; (OR = 1.17, CI = 1.07, 1.28, *p* value = 0.001); region (*p* values < 0.05)

Ettarh [33]	2012	Cross-sectional	Multivariate analysis (hazard ratio)	Country-wide (Kenya)	To compare the influence of geographical location and key maternal factors on under-five deaths	Kenya DHS 2008-2009 (*n* = 16,162 live births)	Place of residence; rural (HR = 3.61, 95%CI = 1.27, 10.32, *p* value < 0.05), age of mother; ≥32 with Ref to 15-20 years (HR = 0.32, CI = 0.17, 0.60, *p* value < 0.01), birth order; 2-3 with Ref to 1 (HR = 2.60, CI = 1.03, 6.58, *p* value < 0.05), ≥4 (HR = 3.77, CI = 1.41, 10.09, *p* value < 0.05), wealth index; middle with Ref to low (HR = 0.74, CI = 0.59, 0.93, *p* value < 0.05), highest (HR = 0.77, CI = 0.58, 0.98, *p* value < 0.05), province/region (*p* value for HR < 0.05), duration of breastfeeding; >12 months with Ref to <6 months (HR = 0.13, CI = 0.02, 0.84, *p* value < 0.05)

Ezeh [25]	2015	Cross-sectional	Cox proportional hazard regression model	Country-wide (Nigeria)	To identify the factors associated with under-five mortality	Nigeria DHS 2003, 2008 and 2013 (*n* = 66,154 live births, combined sample)	Birth order and birth interval; 1^st^ child with Ref to 2^nd^ or 3^rd^ child, interval > 2 years (AHR = 1.42, *p* value < 0.001), 2^nd^ or 3^rd^ child, interval ≤ 2 years (AHR = 1.48, *p* value < 0.001), 4^th^ or higher, interval ≤ 2 years (AHR = 1.89, *p* value < 0.001), household wealth index; middle with Ref to rich (AHR = 1.42, *p* value = 0.001), poor (AHR = 1.43, *p* value = 0.001), mother's education; no education with Ref to secondary or higher (AHR = 1.19, *p* value = 0.032), place of residence; rural (AHR = 1.29, *p* value = 0.001), sex of child; male (AHR = 1.24, *p* value < 0.001), mode of delivery; caesarean with Ref to noncaesarean (AHR = 1.74, *p* value = 0.001), size of child at birth; small or very small with Ref to average or large (AHR = 1.47, *p* value < 0.001), mother's age; less than 20 with Ref to 30-39 (AHR = 1.44, *p* value = 0.004), 40-49 (ARH = 1.47, *p* value < 0.001)

Fenta [23]	2020	Cross-sectional	Two-part random-effects regression model (negative binomial hurdle model)	Country-wide (Ethiopia)	To identify the potential risk factors for child mortality	Ethiopian DHS 2016 (*n* = 14,370 live births)	Child vaccination; yes (IRR = 0.735, 95%CI = 0.647, 0.834), family size; (IRR = 0.968, CI = 0.956, 0.980), age of mother; (IRR = 1.052, CI = 1.047, 1.056), ANC visit; 1-3 with Ref to no (IRR = 0.841, CI = 0.737, 0.960), 4 or above (IRR = 0.814, CI = 0.702, 0.944), previous birth interval; 25-36 months with Ref to ≤24 months (IRR = 0.836, CI = 0.787, 0.889), 37 and above (IRR = 0.728, CI = 0.676, 0.783), use of contraceptive; yes (IRR = 0.885, CI = 0.814, 0.962), father's education; secondary and above with Ref to no education (IRR = 0.695, CI = 0.594, 0.814), mother's education; primary with Ref to no education (IRR = 0.785, CI = 0.713, 0.864), father's occupation; had working with Ref to no (IRR = 1.125, CI = 1.049, 1.206), place of delivery; private sector with Ref to home (IRR = 0.609, CI = 0.405, 0.916), type of births; multiple with Ref to single (IRR = 1.355, CI = 1.249, 1.471), age of mother at first birth; 17 and above with Ref to ≤16 (IRR = 0.711, CI = 0.674, 0.750), birth order; 1-3 with Ref to first (IRR = 1.372, CI = 1.262, 1.491), 4 and above (IRR = 1.487, CI = 1.373, 1.612), religion; Muslim with Ref to orthodox (IRR = 1.255, CI = 1.129, 1.394), between enumeration area (level 2) (IRR = 0.526, CI = 0.474, 0.548)

Ghimire [37]	2019	Cross-sectional	Survey-based Cox proportional hazard model	Country-wide (Nepal)	To identify the factors associated with under-five child mortality	Nepal DHS (2001-2016) (*n* = 16,802 live births)	Previous dead child; yes (AHR = 15.97, 95%CI = 11.64, 21.92, *p* value < 0.001), tetanus toxoids (TT) vaccination during pregnancy; one TT with Ref to two or more TT (AHR = 1.54, CI = 1.09, 2.16, *p* value 0.013), no TT (AHR = 2.39, CI = 1.89, 3.01, *p* value < 0.001), contraceptives use; no (AHR = 2.03, CI = 1.57, 2.62, *p* value < 0.001), ethnicity; Madhesi with Ref to Brahmin/Chhetri (AHR = 1.73, CI = 1.29, 2.32, *p* value < 0.001), mother's literacy level; cannot read with Ref to can read (AOR = 1.33, CI = 1.03, 1.72, *p* value = 0.031), mother's occupation; agriculture with Ref to not working (AHR = 1.45, CI = 1.06, 1.96, *p* value = 0.018), skilled/professional (AHR = 2.15, CI = 1.40, 3.30, *p* value < 0.001), mother's age; 20-29 with Ref to 40-49 years (AHR = 1.88, CI = 1.24, 2.86, *p* value = 0.003), < 20 (AHR = 2.76, CI = 1.57, 4.85, *p* value < 0.001), birth rank and birth interval; 1^st^ child with Ref to 2^nd^/3^rd^ birth rank, >2 years (AHR = 2.55, CI = 1.77, 3.68, *p* value < 0.001), 4^th^/higher birth rank, interval > 2 years (AHR = 0.36, CI = 0.24, 0.52, *p* value < 0.001), 4^th/^higher birth rank, interval ≤ 2 years (AHR = 0.62, CI = 0.42, 0.91, *p* value = 0.015)

Gutema [24]	2022	Open cohort population-based longitudinal surveillance design	Multilevel logistic regression	KHDSS, eastern Ethiopia	To assess under-five mortality focusing on the trends and associated factors based on 2008-2016 data in KHDSS	KHDSS in Kersa district, East Hararghe Zone, Oromia region, Ethiopia (*n* = 18,759 live births)	ANC visits; yes (AOR = 0.61, 95%CI = 0.49, 0.74), education of mother; elementary with Ref to no-education (AOR = 0.58, CI = 0.49, 0.68), birth weight; normal with Ref to low (AOR = 0.78, CI = 0.64, 0.95), big (AOR = 5.16, CI = 1.98, 13.47), window presence in the house; yes (AOR = 0.80, 95%CI = 0.67, 0.95), occupation of mother; employed (AOR = 0.66, 95%CI = 0.48, 0.91), family size; two and/or less with Ref to more than 5 (AOR = 0.37, CI = 0.22, 0.37), 3-4 (AOR = 0.38, CI = 0.31, 0.45)

Hammer [21]	2006	Cross-sectional/cohort	Cox proportional hazards regression	Sub-Saharan Africa (Burkina Faso)	To identify the effect of risk factors for childhood mortality	DSS and DHS (1998-1999) of Burkina Faso (*n* = 6,195 for DSS, *n* = 4,957 for DHS live births)	Birth order; first with Ref to 2-4 (HR = 1.22, 95%CI = 1.04, 1.43, *p* value = 0.02), multiple birth (HR = 3.19, CI = 2.51, 4.05, *p* value < 0.01), family size; 7-10 with Ref to ≤6 (HR = 0.79, CI = 0.69, 0.91, *p* value < 0.01), >11 (HR = 0.66, CI = 0.58, 0.76, *p* value < 0.01), religion; Catholic with Ref to Muslim (HR = 0.84, CI = 0.73, 0.98, *p* value = 0.03), traditional (HR = 1.30, CI = 1.09, 1.54, *p* value < 0.01)

Iddrisu [29]	2020	Cross-sectional	Logistic regression model using frequentist and Bayesian framework	Country-wide (Ghana)	To identify the risk factors of child mortality	Ghana DHS 2014 (*n* = 5,884 live births)	Mode of delivery; caesarean section with Ref to not caesarean section (AOR = 1.449, 95%CI = 1.005, 2.089, *p* value < 0.05), size of child at birth; average with Ref to small (AOR = 0.498, CI = 0.362, 0.684, *p* value < 0.05), large (AOR = 0.513, CI = 0.384, 0.685, *p* value < 0.05), mother's education; formal education with Ref to no formal education (AOR = 0.766, CI = 0.596, 0.984, *p* value < 0.05)

Kandala [34]	2014	Cross-sectional	Logistic regression and multivariate Bayesian geo-additive survival analysis	Country-wide (DR of Congo)	To examine province-level geographic variation in under-five mortality and accounting risk factors of under-five mortality	Congo DHS 2007 (*n* = 8,992 live births)	Preceding birth interval; <24 months with Ref to ≥24 months (AOR = 1.14, 95%CI = 1.04, 1.26), place of delivery; home with Ref to hospital (AOR = 1.13, CI = 1.01, 1.27), marital status of mother; single with Ref to married (AOR = 1.16, CI = 1.03, 1.33)

Kayode [26]	2012	Cross-sectional	Multiple logistic regression	Country-wide (Nigeria)	To determine risk factors of under-five mortality	Nigeria DHS 2008 (*n* = 28,647 live births)	Maternal age; 26-30 with Ref to ≤20 years (OR = 1.70, 95%CI = 1.30, 2.22, *p* value 0.001), 31-35 (OR = 2.48, CI = 1.84, 3.33, *p* value = 0.001), >35 (OR = 2.87, CI = 2.10, 3.91, *p* value = 0.001), maternal age at first marriage; 20-24 with Ref to <15 years (OR = 0.80, CI = 0.70, 0.90, *p* value = 0.001), ≥25 (OR = 0.70, CI = 0.57, 0.85, *p* value = 0.001), use of contraception; traditional with Ref to no method (OR = 0.69, CI = 0.51, 0.85, *p* value = 0.017), health seeking behavior; average with Ref to low (OR = 0.06, CI = 0.05, 0.07, *p* value = 0.001), preceding birth interval; 18-36 with Ref to <18 months (OR = 0.30, CI = 0.26, 0.34, *p* value = 0.001), >36 (OR = 0.09, CI = 0.07, 0.10, *p* value = 0.001), breastfeeding; >18 with Ref to <6 months (OR = 0.43, CI = 0.35, 0.53, *p* value = 0.001), birth order; 2, 3, or 4 with Ref to 1 (OR = 1.93, CI = 1.56, 2.37, *p* value = 0.001), birth weight; small with Ref to normal (OR = 1.31, CI = 1.09, 1.58, *p* value = 0.004), family size; >5 with Ref to 1-5 (OR = 3.54, CI = 3.07, 4.08, *p* value = 0.001), type of toilet; bad toilet with Ref to good toilet (OR = 1.77, CI = 1.46, 2.14, *p* value = 0.001), fuel source; kerosene with Ref to gas (OR = 0.52, CI = 0.44, 0.63, *p* value = 0.001), others (OR = 0.28, CI = 0.23, 0.34, *p* value = 0.001), no. of wives; more wives with Ref to one (OR = 1.47, CI = 1.30, 1.66, *p* value = 0.001), type of residence; rural (OR = 1.53, CI = 1.16, 1.14, *p* value = 0.002)

Motsima [30]	2016	Cross-sectional	Multiple logistic regression	Country-wide (Lesotho)	To determine the factors associated with under-five child mortality	Lesotho DHS -2009 (*n* = 3,999 live births)	Sex of child; female (OR = 0.62, CI = 0.42, 0.91, *p* value = 0.016), type of births; multiple (OR = 2.72, CI = 1.02, 7.23, *p* value = 0.046), breastfeeding duration; 13-18 with Ref to 0-12 months (OR = 0.14, CI = 0.072, 0.27, *p* value < 0.001), 19 and above (OR = 0.02, CI = 0.0064, 0.0684, *p* value < 0.001), source of energy; other with Ref to electricity; (OR = 2.54, CI = 1.32, 4.85, *p* value = 0.005), marital status; formerly married with Ref to married (OR = 2.26, CI = 1.56, 4.37, *p* value < 0.001)

Naz [39]	2021	Cross-sectional	Cox proportional hazards regression	Country-wide (Pakistan)	To examine the effect of socioeconomic status and type of residence on under-five mortality	Pakistan DHS 2017/18 (*n* = 19,190 live births)	Mother's education; educated (HR = 0.75, CI = 0.60, 0.93, *p* value < 0.001), mother's employment; working (HR = 1.25, CI = 1.00, 1.06, *p* value < 0.10), birth spacing; 2-3 with Ref to <2 years (HR = 0.57, CI = 0.46, 0.71, *p* value < 0.001), >3 years (HR = 0.56, CI = 0.45, 0.69, *p* value < 0.001), mother's age at first birth; ≥18 years (HR = 0.79, CI = 0.65, 0.95, *p* value < 0.001), birth size; average with Ref to small (HR = 0.64, CI = 0.51, 0.78, *p* value < 0.001)

Naz [35]	2020	Cross-sectional	Cox proportional hazards model	Country-wide (Sierra Leone)	To point out crucial risk factors of under-five mortality	Sierra Leone DHS 2013 (*n* = 24,742 live births)	Age of mother at first birth; >18 with Ref to ≤18 years (AHR = 0.92, 95%CI = 0.86, 0.98, *p* value < 0.001), sex of child; female (AHR = 0.90, CI = 0.84, 0.96, *p* value < 0.001), number of children in the house; 3-4 child with Ref to 1-2 child (AHR = 0.40, CI = 0.34, 0.46, *p* value < 0.001), 5 and above (AHR = 0.35, CI = 0.28, 0.41, *p* value < 0.001), birth interval; >3 years with Ref to <2 years (AHR = 0.70, CI = 0.49, 0.98, *p* value < 0.05), size of child at birth; smaller than average with Ref to very small (AHR = 0.56, CI = 0.47, 0.66, *p* value < 0.001), average or larger (AHR = 0.55, CI = 0.46, 0.65, *p* value < 0.001)

Ogbo [36]	2019	Cross-sectional	Cox proportional hazards model	Country-wide (Tanzania)	To investigate the trends and determinants of neonatal, postneonatal, infant, child, and under-five mortalities in Tanzania from 2004 to 2016	Tanzanian DHS 2004-2005, 2010, 2015-2016 (*n* = 25,951 live births, combine of three Tanzanian DHS)	Type of residence; rural (AHR = 0.79, 95%CI = 0.67, 0.93), mother's education; primary with Ref to secondary or higher (AHR = 1.38, CI = 1.06, 1.80), birth rank and birth interval; first child with Ref to 2 or 3 child, interval > 2 (AHR = 1.39, CI = 1.18, 1.63), 2 or 3 child, interval ≤ 2 (AHR = 1.43, CI = 1.14, 1.79), 4 or more child, interval ≤ 2 (AHR = 1.58, CI = 1.27, 1.98), sex of child; male (AHR = 1.21, CI = 1.07, 1.37), size of child at birth; small or very small with Ref to average or larger (AHR = 1.90, CI = 1.59, 2.27)

Rhoda [27]	2019	Cross-sectional descriptive study	Binary logistic regression	Federal capital territory of Nigeria	To examine the effect of demographic and socioeconomic characteristics of women to under-five child mortality	Primary data (*n* = 200 live births)	Education of mother (beta coefficient = 34.44, *p* value = 0.019), main occupation of mother (beta coefficient = 274.48, *p* value = 0.005), ethnic group (beta coefficient = 617.81, *p* value = 0.091), average monthly income of mother (beta coefficient = 300.42, *p* value = 0.064)

Vanthy [40]	2019	Cross-sectional	Weibull hazards regression	Country-wide (Cambodia)	To define persistent and emerging factors associated with under-five mortality in Cambodia	Cambodian DHS 2010 and 2014 (*n* = 8232 Cambodian DHS 2010, *n* = 7,165 Cambodian DHS 2014)	Type of birth; twin (CDHS 2010: AHR = 2.08, 95%CI = 1.05, 4.13), birth interval; 2-3 years with Ref to <2 years (CDHS 2010: AHR = 0.49, CI = 0.32, 0.76; CDHS 2014: AHR = 0.48, CI = 0.24, 0.95), more than 3 years (CDHS 2010: AHR = 0.59, CI = 0.41, 0.86; CDHS 2014: AHR = 0.47, CI = 0.25, 0.87), age of mother at child birth; more than 40 years with Ref to <20 years (CDHS 2010: AHR = 3.55, CI = 1.80, 7.03; CDHS 2014: AHR = 3.21, CI = 1.13, 9.08), mother's education; primary with Ref to no education (CDHS 2010: AHR = 1.41, CI = 1.04, 1.91), secondary or higher (CDHS 2010: AHR = 1.86, CI = 1.16, 2.97; CDHS 2014: AHR = 1.95, CI = 1.05, 3.62), place of residence; rural CDHS 2014: AHR = 2.99, CI = 1.28, 6.97), region; plain with Ref to Phnom Penh (CDHS 2010: AHR = 2.92, CI = 1.15, 7.39), ANC visit; have ANC (CDHS 2010: AHR = 0.42, CI = 0.29, 0.62; CDHS 2014: AHR = 0.33, CI = 0.18, 0.59), TT vaccination; received >2 dose with Ref to not received at last birth (CDHS 2010: AHR = 0.66, CI = 0.45, 0.97), child vaccination status; not fully immunized with Ref to fully immunized (CDHS 2010: AHR = 01.64, CI = 1.40, 1.93; CDHS 2014: AHR = 3.90, CI = 3.13, 4.86)

Yaya [31]	2018	Cross-sectional	Multivariable Cox proportional hazards regression	Multicountry analysis (five sub-Saharan African countries-Chad, Demographic Republic (DR) of Congo, Mali, Niger, and Zimbabwe)	To examine the maternal factors associated with under-five mortality	DHS data from five sub-Saharan Africa countries (*n* = 40,754 live births)	Age of mother at first birth; (Mali: HR = 1.07, 95%CI = 1.05, 1.09, *p* value < 0.05; Zimbabwe: HR = 1.07, CI = 1.03, 1.09, *p* value < 0.05), place of residence; rural (Chad: HR = 1.11, CI = 1.01, 1.19; DR Congo: HR = 1.29, CI = 1.02, 1.57; Mali: HR = 1.28, CI = 1.01, 1.64; Niger: HR = 1.14, CI = 1.01, 1.33; Zimbabwe: HR = 1.01, CI = 0.83, 1.20, *p* value < 0.05), education of mother; secondary with Ref to no formal (Zimbabwe: HR = 0.62, CI = 0.38, 0.99, *p* value < 0.05; higher: HR = 0.47, CI = 0.23, 0.96, *p* value < 0.05), wealth index; richer with Ref to poorest (DR Congo: HR = 0.89, CI = 0.79, 0.99, *p* value < 0.05), richest (DR Congo: HR = 0.78, CI = 0.65, 0.94; Niger: HR = 0.84, CI = 0.61, 0.95, *p* value < 0.05), marital status; not currently married with Ref to currently married/in union (DR Congo: HR = 1.24, CI = 1.11, 1.40; Mali: 2.43, CI = 1.63, 3.64; Niger: HR = 1.59, CI = 1.24, 2.30; Zimbabwe: HR = 1.33, CI = 1.06, 1.67, *p* value < 0.05), type of birth; multiple (Niger: HR = 1.14, CI = 1.04, 1.31; Zimbabwe: HR = 1.19, CI = 1.01, 1.57, *p* value < 0.05), mode of delivery; caesarean section (Chad: HR = 1.32, CI = 1.00, 1.77; DR Congo: HR = 1.20, CI = 1.01, 1.43; Mali: HR = 1.42, CI = 1.08, 1.85; Niger: HR = 1.43, CI = 1.06, 1.92; Zimbabwe: HR = 1.49, CI = 1.03, 2.15; *p* value < 0.05), size of child; small with Ref to large (DR Congo: HR = 1.13, CI = 1.02, 1.19; Niger: HR = 1.15, CI = 1.02, 1.22, *p* value < 0.05), birth order; 5 and above (Chad: HR = 0.44, CI = 0.40, 0.49; DR Congo: 0.44, CI = 0.39, 0.48, *p* value < 0.05), birth interval; 18-24 with Ref to <18 (DR Congo: HR = 0.85, CI = 0.74, 0.97), >24 (Chad: HR = 0.88, CI = 0.79, 0.98; DR Congo: HR = 0.85, CI = 0.75, 0.95, *p* value < 0.05)

OR: odds ratio; RR: risk ratio; AOR: adjusted odds ratio; CI: confidence interval; HR: hazard ratio; AHR: adjusted hazard ratio; IRR: incidence rate ratio; Ref: reference category; DHS: demographic and health survey; DSS: demographic surveillance system; KHDSS: Kersa health and demographic surveillance site.

**Table 2:** Thematic distribution of factors associated to under-five child mortality.

Thematic factors	Factors associated with under-five child mortality	*n* (%)
Maternal factors	Marital status, age of mother at childbirth, working status of mother, age of mother at first birth, ethnic group, religion, duration of breastfeeding, mother's education, age at first marriage, average monthly income of mother, main occupation of mother, delivery complication, smoking habit	13 (27.7)
Socioeconomic factors	Type of fuel for cooking, distance traveled for water, source of drinking water, type of toilet, type of floor, type of roof of house, household's income/wealth index, family size, no. of children under age 5 at home, distance to health center, type of wall of house, window presence in house, no. of wives	13 (27.7)
Child-related factors	Type of birth, birth order, sex of child, size of child at birth, birth order and birth interval, previous death of sibling, birth interval, no. of children at house	8 (17)
Utilization of health care-related factors	Place of delivery, child vaccination, ANC visits, use of contraceptive, TT vaccination during pregnancy, mode of delivery, health seeking behavior, birthplace and mode of delivery	8 (17)
Community level factors	Place of residence, region, variations between enumeration areas	3 (6.4)
Paternal factors	Father's education, occupation of father	2 (4.2)

## Supplementary Materials

Supplementary Materials The supplementary information associated with this study contain results of NIH quality assessment tools employed to evaluate the study. Each study was appraised using fourteen different tools, and outcomes are summarized in a supplementary table 1.
